# Label-free multimodal electro-thermo-mechanical (ETM) phenotyping as a novel biomarker to differentiate between normal, benign, and cancerous breast biopsy tissues

**DOI:** 10.1186/s13036-023-00388-y

**Published:** 2023-11-13

**Authors:** Anil Vishnu G. K., Gayatri Gogoi, Midhun C. Kachappilly, Annapoorni Rangarajan, Hardik J. Pandya

**Affiliations:** 1 Center for BioSystems Science and Engineering, Indian Institute of Science, Bangalore, Karnataka 560012 India; 2 Department of Pathology, Assam Medical College, Dibrugarh, Assam 786002 India; 3 Department of Electronic Systems Engineering, Indian Institute of Science, Bangalore, Karnataka 560012 India; 4 Department of Developmental Biology and Genetics, Indian Institute of Science, Bangalore, Karnataka 560012 India; 5 Centre for Product Design and Manufacturing, Indian Institute of Science, Bangalore, Karnataka 560012 India

**Keywords:** Biophysical phenotyping, Breast cancer, Diagnostics, Microsensors, Gaussian process

## Abstract

**Background:**

Technologies for quick and label-free diagnosis of malignancies from breast tissues have the potential to be a significant adjunct to routine diagnostics. The biophysical phenotypes of breast tissues, such as its electrical, thermal, and mechanical properties (ETM), have the potential to serve as novel markers to differentiate between normal, benign, and malignant tissue.

**Results:**

We report a system-of-biochips (SoB) integrated into a semi-automated mechatronic system that can characterize breast biopsy tissues using electro-thermo-mechanical sensing. The SoB, fabricated on silicon using microfabrication techniques, can measure the electrical impedance (Z), thermal conductivity (K), mechanical stiffness (k), and viscoelastic stress relaxation (%R) of the samples. The key sensing elements of the biochips include interdigitated electrodes, resistance temperature detectors, microheaters, and a micromachined diaphragm with piezoresistive bridges. Multi-modal ETM measurements performed on formalin-fixed tumour and adjacent normal breast biopsy samples from *N* = 14 subjects were able to differentiate between invasive ductal carcinoma (malignant), fibroadenoma (benign), and adjacent normal (healthy) tissues with a root mean square error of 0.2419 using a Gaussian process classifier. Carcinoma tissues were observed to have the highest mean impedance (110018.8 ± 20293.8 Ω) and stiffness (0.076 ± 0.009 kNm^−1^) and the lowest thermal conductivity (0.189 ± 0.019 Wm^−1^ K^−1^) amongst the three groups, while the fibroadenoma samples had the highest percentage relaxation in normalized load (47.8 ± 5.12%).

**Conclusions:**

The work presents a novel strategy to characterize the multi-modal biophysical phenotype of breast biopsy tissues to aid in cancer diagnosis from small-sized tumour samples. The methodology envisions to supplement the existing technology gap in the analysis of breast tissue samples in the pathology laboratories to aid the diagnostic workflow.

**Supplementary Information:**

The online version contains supplementary material available at 10.1186/s13036-023-00388-y.

## Background

Among cancers affecting women, breast cancer accounted for nearly 25% of all new cancer cases and 15% of cancer-related mortality in 2020 [1]. The accurate diagnosis and timely therapeutic intervention (surgery, radiation, or chemotherapy) of breast cancer have significantly improved the 5-year survival rates [2]. While histopathology and immunohistochemistry are the current standards for the diagnosis of breast cancer, technologies that can provide a quick, preliminary assessment of the nature of the tumour (benign or malignant) in extracted biopsy sample has potential utility in the pathology labs and surgical margin assessment and planning [3, 4].

While the identification of malignant tumours is of primary importance in diagnostics, the ability to also detect benign tumours has significance in deciding the nature of surgery to be performed [5]. If the preoperative diagnosis is benign, the tumours are usually removed at the behest of the patient. In such cases, the target is to remove only the tumour with a minimal amount of surrounding healthy tissue to minimize cosmetic damage and disfigurement [6]. When the preoperative diagnosis is malignant, the aim of the surgical procedure is therapeutic. In such a scenario, the target is to ensure that no malignant lesions are left behind so as to reduce the risk of relapse [7]. This requires the excision of an additional margin of the adjacent normal tissue around the malignant tumour. During surgery, tissue biopsies are often analyzed using frozen section examination to arrive at the real-time estimate of the boundary for the surgical excision [8]. The ability to classify the sample as benign, malignant, or healthy, thus, has implications for surgical outcomes and patient survival.

The biophysical properties of the breast tissue, such as its thermal response, electrical conductivity, and stiffness, have been used to develop several non-invasive cancer screening technologies such as mammography, ultrasonography, thermography, and optical coherence tomography, to name a few [9, 10]. In the context of analyzing *ex-vivo* biopsy samples during diagnosis and surgery, assessing the biophysical properties of the biopsy tissues requires significantly lesser sample preparation steps than routine biochemical analysis [11, 12]. Several aspects of tumourigenesis and tumour progressions, such as the increased proliferation of cells, smaller volume fraction of intact cells, leaky blood vessels, and remodeling of the extracellular matrix (ECM), have been shown to alter the electrical, thermal, and mechanical properties of cancer tissues, including in breast cancer [13–16]. For the samples of small dimensions that are often extracted during surgical procedures, technologies for biophysical phenotyping of the tissue biopsies ex vivo are required to be miniaturized.

**Fig. 1:**
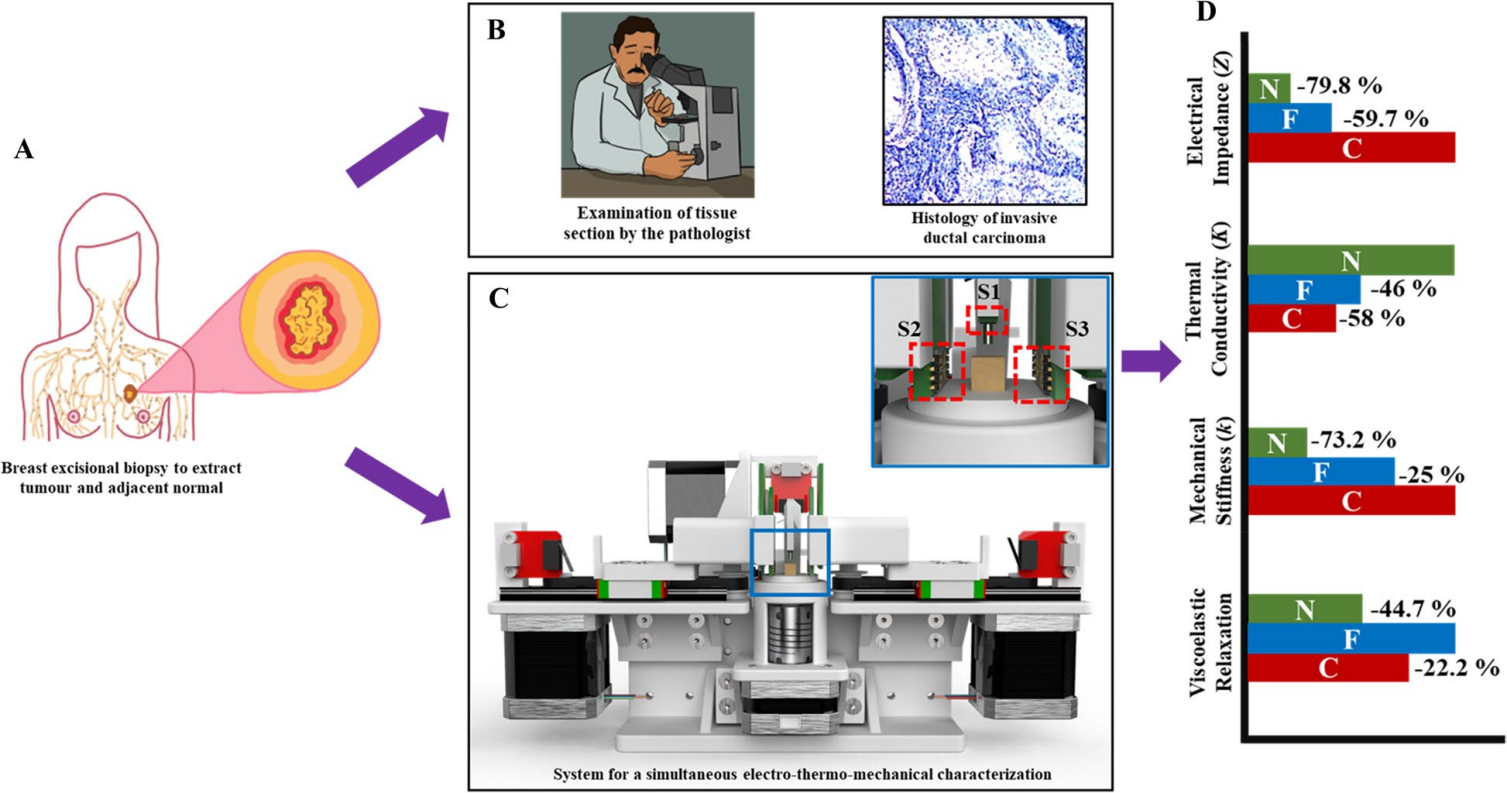
Scheme of the study showing **A** breast excisional biopsy, **B** histopathological examination of the sample to identify malignancy, **C** system for simultaneous electro-thermo-mechanical phenotyping of the biopsy tissue with the system-of-biochips for force sensing and electro-thermal sensing attached to the tissue probes shown in inset, and **D** summary of key results from the study comparing tumour and normal based on the results from the electrical, thermal, and mechanical characterization

Previously our group has reported the observation of scaling laws in the temperature and frequency-dependent electrical transport through ex vivo breast biopsy samples and employed the scaling law parameters to classify the sample as a normal or tumour [17]. We also observed that the bulk DC electrical resistivity measured at an elevated tissue temperature of 37 ℃, when combined with a concordant measurement of the thermal conductivity, was also able to differentiate between adjacent normal and tumour with a high level of statistical significance [18]. However, these studies only classified the samples as normal or tumour and did not look at differences between benign and malignant tumours. In this work, a system of biochips (SoB) integrated with the semi-automated system is reported that can perform the electrical, thermal, and mechanical (ETM) characterization of breast biopsy tissue. The platform enables the measurement of the electrical impedance (Z), the thermal conductivity (*K*), mechanical stiffness (*k*), and viscoelastic relaxation (R) towards a comprehensive biophysical understanding of the sample. We observe that such a multi-modal electro-thermo-mechanical phenotyping can serve as a novel biomarker to differentiate between adjacent normal, benign, and cancerous tissues with a high level of statistical significance. The scheme of the study is summarized in Fig. 1.

## Results

### Semi-automated experimental setup integrated with a system-of-biochips (SoB)

**Fig. 2:**
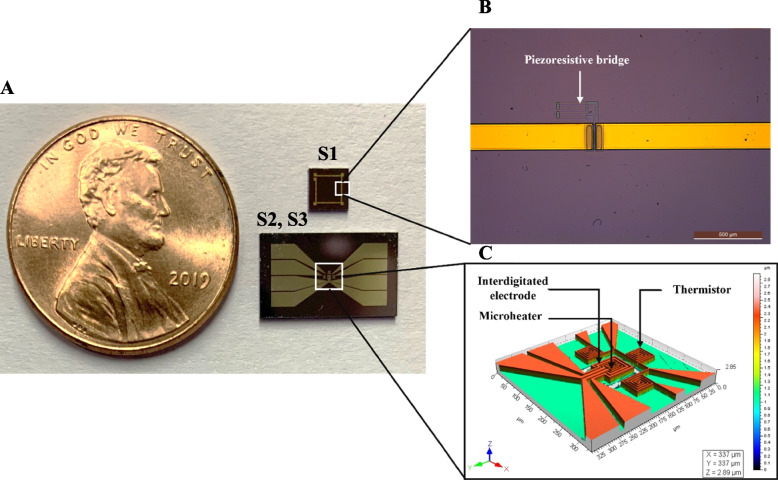
System-of-biochips (SoB) for electro-thermo-mechanical phenotyping of breast tissues. **A **optical photograph of the biochips, **B** optical microscope image showing the piezoresistive bridge in the force sensor for mechanical characterization, and **C** optical profilometry image of the electro-thermal biochip showing the microheater, interdigitated electrodes and thermistor

The experimental setup consists of the schematic shown in Fig. 1C which is a semi-automated system with two horizontal probes (X-direction) and one vertical probe (Z-direction) for interacting and taking measurements from the tissue sample. The system integrates three biochips, namely S1, S2, and S3 (as shown in Fig. 1C inset), which constitutes the system of biochips (SoB). S1 acts as the microforce sensor for mechanical characterization, while S2 and S3 are used for electro-thermal characterization. The photograph of the fabricated biochips is shown in Fig. 2A. The design of S1 consists of four piezoresistive bridges fabricated on a thin diaphragm created on a silicon substrate. The dimension of S1 is 2.5 mm x 2.5 mm. The optical microscope image of one piezoresistive bridge is shown in Fig. 2B. The functional elements of S2 and S3 consist of a microheater, interdigitated electrodes, and thermistors around the microheater. While the microheater is used to heat the tissue, the interdigitated electrodes and the thermistors measure the electrical and thermal properties of the tissue placed between S2 and S3. A square shape profile is provided for the microheater, thermistors, and the electrodes to geometrically match with the cuboidal shape of the sample tissues, which makes subsequent data analysis and parameter extraction simpler. The biochip has an overall dimension of 7 mm x 12 mm with an active area of 1 mm x 1 mm. An optical profilometry image of the sensing elements is shown in Fig. 2C.

**Fig. 3:**
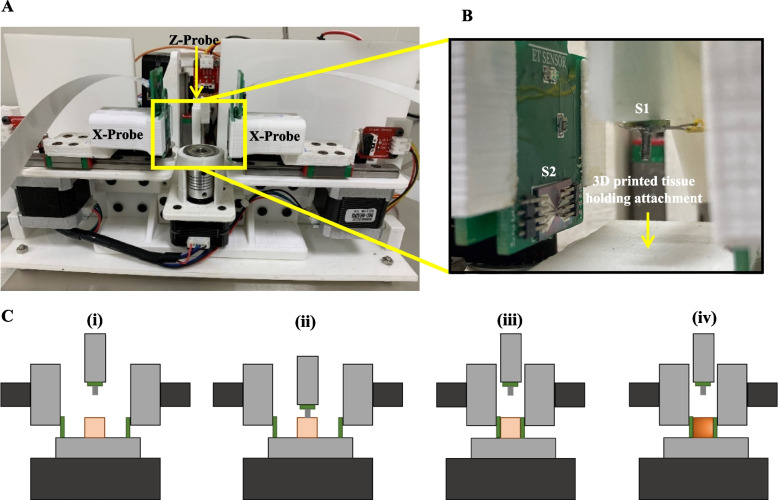
Experimental setup. **A** Photograph of the semi-automated system with the three probes and system of biochips, **B** close-up view of one electrothermal biochip and the force sensing chip connected to the probes, and **C** experimental protocol for the ETM measurements (i) sample is loaded into the system, (ii) S1 indents the tissue followed by stress-relaxation for 150s, (iii) S1 retracts, S2 and S3 probe the tissue, make contact, and heats the sample to 37 ℃, and (iv) measurements are captured from the RTDs followed by the impedance characterization

The photograph of the assembled system with the probes, electronic modules, biochips, and rotary platform for placing the sample is shown in Fig. 3A. The biochip S1, which integrates the force sensor, is wire bonded on a carrier printed circuit board and attached to the vertical probe along with a force transfer mesa, while the biochips with the electro-thermal sensors, S2 and S3, are attached to the two horizontal probes. This arrangement is shown schematically in the inset of Fig. 1C, and the actual photograph of the arrangement is shown in Fig. 3B. The probes are actuated through NEMA-17 stepper motors connected to a microcontroller and motor driver system. The biochips are connected to the electronic modules in the system using flexible flat cables (FFC). The electronic modules integrate the circuits for multiplexing and measuring the voltages from the force sensor and the resistances from the thermistors and interdigitated electrodes. The microheater on S2 is connected to a constant current voltage driver circuit to act as the heat source for the thermal characterization of the tissue. The microheater structure on S3 acts as a thermistor to detect the heat transmitted through the tissue, along with the other three thermistors around it. Finally, the interdigitated electrodes on S2 and S3 are connected externally to the GW-INSTEK LCR 8105G impedance analyzer system for electrical measurements. The complete actuations and measurements are controlled through a laptop connected to the system through the microcontroller via the UART port through serial communication. Before loading the samples, a disposable 3D printed attachment for holding the tissue surface is placed on the rotary platform to avoid contamination of the setup (shown in Fig. 3B).

### Protocol for electro-thermo-mechanical characterization of breast biopsy tissues

The experiments are conducted in a class 10000 clean room with a controlled ambient temperature of 21 ℃. The experimental methodology of evaluating the system involves capturing the electrical, thermal, and mechanical properties of the tissue sample loaded into the system using the SoB. The Z-axis probe indents the sample vertically and captures the mechanical force response and stress relaxation using the microforce sensor attached to it. The mesa interacts with the tissue and transfers the force to the diaphragm in the sensor S1, and the changes in the piezoresistive bridges are recorded by the onboard electronics through the wires attached to the carrier PCB. The two X-axis probes capture the electro-thermal properties of the sample, such as its electrical impedance and thermal conductivity, using the biochips attached to them. The microheater on S2 heats the tissue to the required temperature, and the interdigitated electrodes between S2 and S3 capture the electrical impedance data across the tissue sample. This methodology is chosen so that once the sample is loaded into the system, its complete biophysical phenotype (electrical, thermal, and mechanical) can be captured. The mechanical characterization, which involves loading the sample to 30 mN force followed by stress-relaxation and unloading to the original state, is performed first as the electro-thermal characterization involves heating the tissue sample to 37 ℃. Once the sample is loaded into the system, the following protocol is used to capture the ETM properties (also shown graphically in Fig. 3C (i to iv):

1. The Z-axis probe with the force sensor indents the sample at a constant rate of 30 μm/s to a compressive load of 30 mN. The sample is kept loaded for 150 s to capture the relaxation data, and then the Z-axis probe is moved up to unload the sample. The load-displacement data is captured from the force sensor.
2. After this, the two X-axis probes, with the biochips for electro-thermal characterization, approach the sample at 20 μm/s and make contact.
3. The microheater on S2 is switched on and is used to heat the tissue to a temperature of 37 ℃ in steps of 3 ℃. At each temperature point, the resistance values of the thermistors are recorded. These resistance values are then mapped to the sensed temperature and used to compute the thermal conductivity.
4. At 37 ℃, the impedance of the sample from 10 Hz to 3 MHz at 100 mV excitation voltage is captured across the IDEs on the two biochips.
5. After the impedance and thermistor measurements at 37 ℃ are completed, the X-axis probes move away from the sample, thereby completing the measurements.

A representative video showing the system in operation is provided as a Supplementary Video. To clearly indicate the clearances between the probes, this video shows a position where all probes are in contact with the tissue. For the actual experiments, the Z-axis probe indents the tissue and moves back, followed by the measurements using the X-axis probes (as detailed in Fig. 3C). The video captured with a larger-sized tissue for clear visibility of the different components is shown.

### Experimental results and biophysical parameters from the ETM characterization

**Fig. 4:**
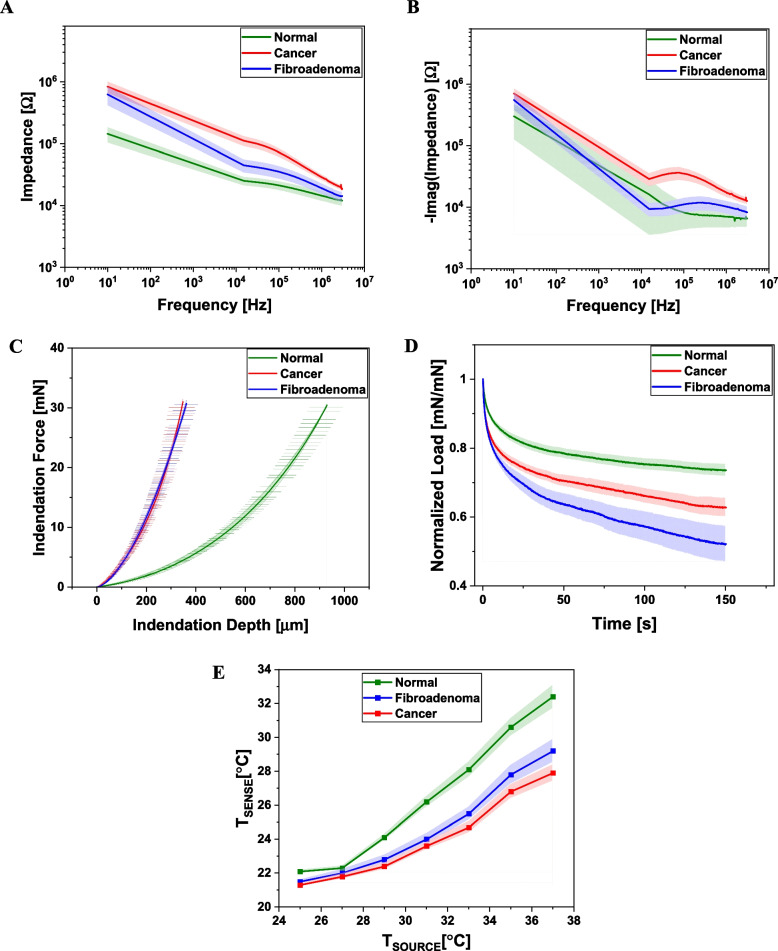
Mean plots of ETM measurements from *N* = 14 paired breast biopsy samples. **A** and **B** shows the magnitude of the complex impedance and imaginary component of the complex impedance as a function of frequency, **C** the mechanical loading characteristics obtained for the samples, **D** viscoelastic relaxation curves and **E** the thermal response of the samples to heating showing the temperature at source and sense points

The electrical impedance at 37 ℃ as a function of frequency, the conducted thermal energy through the tissue, and the mechanical loading and relaxation response are the electrical (E), thermal (T), and mechanical (M) measurements, respectively, performed on each sample. Figure 4 shows the summary of the ETM characterization measurements of the samples from *N* = 14 subjects indicating the mean curves with error bands for the three sample groups, viz. adjacent normal (AN), fibroadenoma (FA), and carcinoma (CA). Since the experiment protocol involves heating the tissue, which might cause irreversible changes, measurements were not repeated on the same sample. However, for each of the 10 carcinoma, 4 fibroadenoma, and 14 adjacent normal tissue samples, two samples each were extracted and measured. Thus, experiments were conducted from a total of *n* = 56 samples from *N* = 14 subjects. Additionally, to quantify the variations between experiments, the electrical impedance and mechanical characterization experiments without heating the tissue were carried out on a few samples multiple times (*n* = 3). The maximum coefficient of variation observed was 6.5%.

Figure 4 A and B show the mean plots of the magnitude and imaginary part of the complex electrical impedance as a function of frequency measured at a tissue temperature of 37 ℃. The impedance magnitude plots of each of the 14 sample pairs are shown in Fig. S1. The plot of the mean phase response and the plot of the real part of the impedance vs. the imaginary part for the AN, CA, and FA groups is also shown in Fig. S2. The mean impedance of CA is observed to be higher than AN and FA at all the measured frequencies. The mean plot of the imaginary part of the impedance (Fig. 4B) has a continuously decreasing trend for the AN group, while for the FA and CA samples, there is a kink in the low-frequency region. However, the trend of higher mean impedance for the CA group compared to the AN and FA is also observed for the imaginary part.

The impedance curve data for the sample groups also facilitates the extraction of circuit parameters which can provide insights into the tissue organization. The standard cole-cole model is modified with two capacitances, C_dl,_ to account for the double layer capacitance at the tissue-electrode interface at both ends of the sample. The other circuit parameters of the tissue are similar to the Cole-Cole model, namely, the extracellular resistance R_e_, the membrane resistance R_m_, the membrane capacitance C_m_, and the intra-cellular resistance R_i_. The equivalent circuit used to fit the mean impedance curves is shown in Fig. S3. The fitted curves for the mean impedance and the mean phase response along with the experimental data for the three groups, are shown in Figs. S4 and S5, respectively. The CA samples had significantly (*p* < 0.001) higher R_e_ (1.75e5 ± 7.07e3 Ω) and R_i_ (3.29e4 ± 1.49e3 Ω) than FA (R_e_ = 3.51e4 ± 4.34e3 Ω; R_i_ = 1.3e4 ± 2.33e2 Ω) and AN (R_e_ = 2.27e4 ± 1.78e3 Ω; R_i_ = 1.23e4 ± 5.31e2 Ω). R_m_ was calculated to be highest for the FA (5.48e6 ± 1.14e5 Ω, *p* < 1e-5) samples and similar for CA (6.43e4 ± 3.14e3 Ω) and AN (9.27e4 ± 1.33e4 Ω). The membrane capacitance, C_m_ was calculated to be highest for the CA (1.02e-10 ± 1.77e-11 F, *p* < 0.05) samples while it was similar for the FA (1.11e-11 ± 6.94e-13 F) and AN (1.1e-11 ± 1.63e-12 F) groups. All the fitted circuit parameter values are summarized in Table S1.

The mechanical loading and stress relaxation tests plots for the three groups are shown in Fig. 4C and D, respectively. Figure 4C shows the changes in the indentation force measured by the force sensor, S1, with the indentation depth of the tissue, with the loading force capped to a maximum of 30 mN. The error bars indicate the family of loading curves for each sample within the sample groups. The data for each sample pair is provided in Fig. S6. It can be seen that the mean loading curves for the CA and FA samples overlap each other and are steeper than the AN sample, indicating higher stiffness. The AN samples get indented to a larger extent (larger amount of strain) for the same applied force of the indenter. Figure 4D plots the relaxation in the normalized load with time up to 150 s. It can be seen that the FA group has the maximum relaxation, followed by CA and AN. While in the loading curves, the CA and FA groups were observed to behave in a similar manner, the relaxation curves revealed differences between the two groups, suggesting the utility of such a characterization.

Figure 4E summarizes the results of the thermal characterization of the samples. The x-axis plots the temperature at the source point of heating of the samples using the on-chip microheater in S2. The samples were heated from room temperature to 37 ℃. Given that in-vitro collagen is thermally unstable beyond 37 ℃ [19], the samples were not heated beyond this temperature to avoid any irreversible damage or charring of the tissue that could confound the impedance measurements at 37 ℃. The y-axis plots the temperature detected by the thermistors in S3 from the heat transmitted through the tissue. It can be seen that the AN samples attain a higher temperature at the sense point for a given source temperature than the CA and FA groups, indicating higher thermal conductivity. The CA and FA groups attain similar temperatures at the sense point for the given source temperature, with the CA group having a comparatively lower value than the FA group.

**Fig. 5:**
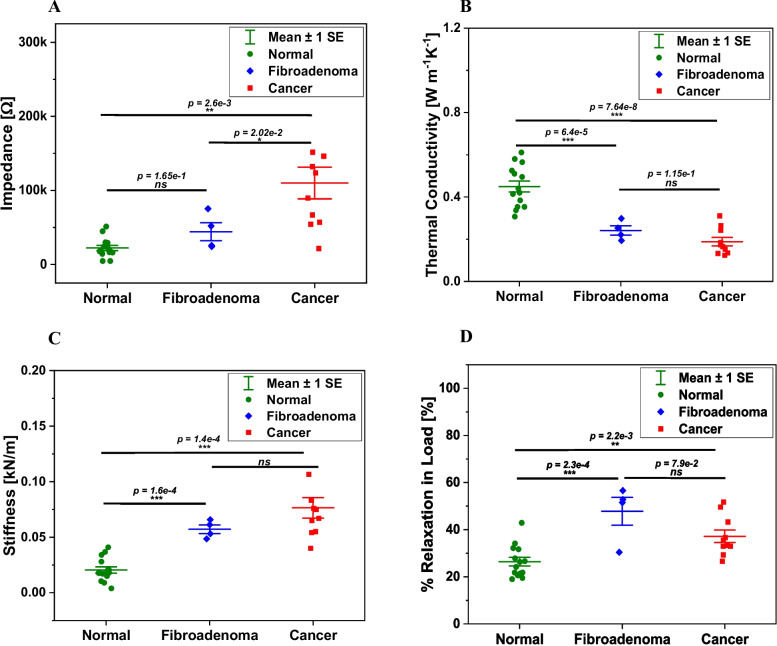
Biophysical parameters extracted from the ETM measurements. **A** impedance at 15 kHz and 37 ℃, **B** thermal conductivity, *K*, **C** mechanical stiffness, *k*, and **D** percentage relaxation in load

From the experimental data in Fig. 4, four key biophysical parameters were extracted. These are namely, the impedance $Z$ (Ω) at 15 kHz, the thermal conductivity $K$ (Wm^-1^ K^-1^) computed at 37 ℃, the mechanical stiffness $k$ (kN/m), and the percentage relaxation in normalized load, $\%R$ (in %). These extracted parameters for each of the three sample groups are shown as scatter interval plots in Fig. 5. All the scatter plots were first assessed for their conformity to the normal distribution using the Shapiro-Wilk test and were observed to follow the distribution. The frequency of 15 kHz was chosen as the non-zero frequency, which showed the highest magnitude of difference between the three groups in Fig. 4A. The thermal conductivity, $K$ of the samples, were computed at 37 ℃ from the mean resistance measured by the thermistors, power input provided to the microheater, and the geometry of the samples. The stiffness, $k$ was calculated as the slope of the loading curves for each sample at 20% strain. The percentage relaxation, $\%R$ value was selected as the endpoint relaxation at 150 s of the stress relaxation experiments.

 From Fig. 5A, the CA group had a significantly higher mean impedance of 110018.8 ± 20293.8 Ω than FA (44261.8 ± 10496.2 Ω, *p* = 2.02e-2) and AN (22206.2 ± 3400.7 Ω, *p* = 2.6e-3). The impedances of the FA and AN groups were not found to be significantly different from each other. The AN group had a significantly higher mean thermal conductivity of 0.45 ± 0.025 Wm^-1^ K^-1^ compared to the FA (0.242 ± 0.019 Wm^-1^ K^-1^, *p* = 6.4e-5) and CA (0.189 ± 0.018 Wm^-1^ K^-1^, *p* = 7.64e-8) groups (Fig. 5B). The $K$ of FA and CA were not significantly different. The CA samples had the highest mean stiffness of 0.076 ± 0.009 kN/m, which was significantly higher (*p* = 1.4e-4) than the AN (0.02 ± 0.003 kN/m) (Fig. 5C). The FA samples were also significantly stiffer than the AN with $k$ of 0.057 ± 0.003 kN/m (*p* = 1.6e-4), which was statistically similar to the CA group. Finally, the FA samples had the highest relaxation in normalized load among the three groups of 47.8 ± 5.1% compared to CA (37.2 ± 2.5%) and AN (26.4 ± 1.75%) (Fig. 5D). The percentage relaxation observed for the FA and CA groups was significantly higher than in the AN sample (*p* < 0.01). The extracted biophysical parameter values from each of the 14 sample pairs are summarized in Table 1.

**Table 1:** Extracted biophysical parameters from *N*=14 paired samples

**SN**	**ADJACENT NORMAL**				**TUMOR**				
	**Impedance** **(Z) [Ω]**	**Thermal Conductivity (K) [Wm**^**-1**^K^**-1**^**]**	**Stiffness (** ***k*** **) [kNm** ^**-1**^ **]**	**Viscoelastic relaxation (%)**	**Type**	**Impedance (Z) [Ω]**	**Thermal Conductivity (K) ** **[Wm** ^**-1**^ **K** ^**-1**^ **]**	**Stiffness (** ***k*** **) [kNm** ^**-1**^ **]**	**Viscoelastic relaxation (%)**
S1	4608.1	0.307	0.034	27.83	CA	21567	0.124	0.055	33.33
S2	29845	0.353	0.00894	20.6	CA	256510	0.174	0.0833	33
S3	24092	0.337	0.01707	24.2	CA	66865	0.184	0.142	29.3
S4	16093	0.353	0.0039	26.6	CA	123700	0.165	0.076	36.6
S5	18394	0.414	0.0178	32.2	CA	57090	0.152	0.075	43.3
S6	21633	0.384	0.015	21.4	CA	146280	0.135	0.067	33
S7	51204	0.44	0.03668	31.7	CA	151700	0.265	0.065	51.7
S8	14276	0.51	0.0104	21.8	CA	89743	0.133	0.1066	49.6
S9	29218	0.42	0.01774	26.3	FA	75189	0.298	0.04856	30.4
S10	17931	0.495	0.0205	19.5	CA	132300	0.242	0.0542	35.5
S11	44780	0.58	0.028	34.1	FA	51961	0.253	0.0612	51.5
S12	17689	0.525	0.0178	19	FA	24258	0.221	0.0531	52.8
S13	16500	0.611	0.0409	42.9	FA	25639	0.194	0.0658	56.6
S14	4624	0.565	0.018	21.8	CA	54433	0.311	0.04	26.6

### Analysis with a combination of the biophysical parameters

As is evident from Fig. 5, no single biophysical parameter extracted from the experimental data is able to differentiate between all the three sample groups (AN, FA, and CA) with statistical significance. The electrical impedance at 15 kHz (Z) is able to differentiate CA from AN and FA, but not AN from FA. Likewise, the thermal conductivity (K), stiffness (k), and percentage relaxation in load (%R) are able to differentiate AN from FA and CA, but not FA from CA. Fisher’s combined probability test was applied to understand whether an analysis with a combination of parameters could differentiate between all the three groups. Before applying the test, each parameter was pairwise tested for its independence to assess whether they add new information that enables differentiation with higher statistical significance.

**Fig. 6:**
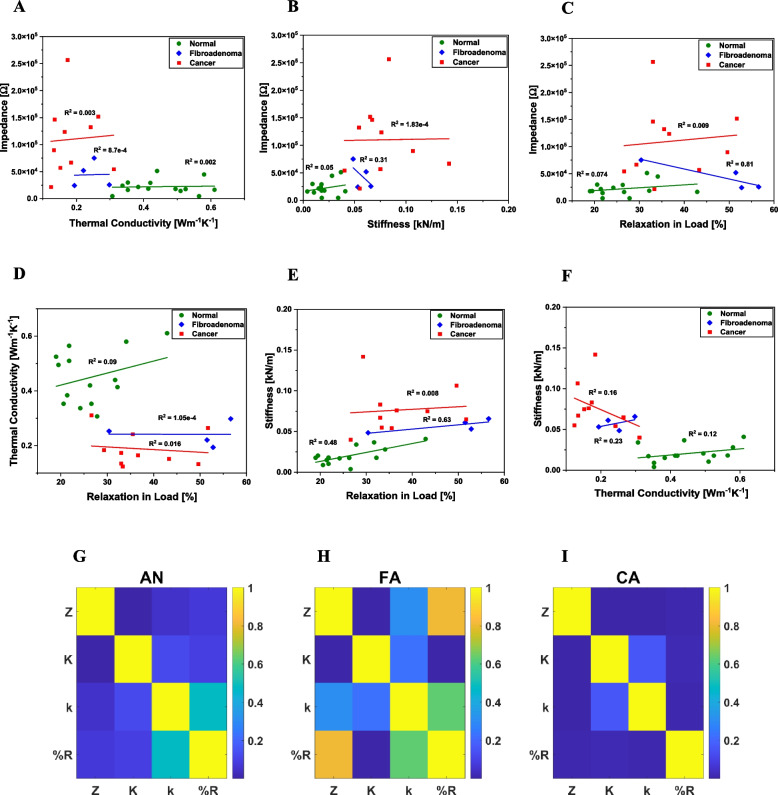
Evaluation of independence of the modalities. **A**–**F** shows the 2D scatter plot of the pair-wise independence plots between the four modalities, namely, impedance at 37 ℃ (Z), thermal conductivity (K), stiffness (k), and percentage relaxation in normalized load (%R), and (**G**), (**H**) and (**I**) show the heatmap of the R-square values indicating the level of independence between the modalities for each class of samples, namely, adjacent normal (AN), fibroadenoma (FA), and carcinoma (CA)

The pairwise testing was performed by plotting each sample on a 2D space defined by two of the four parameters and then repeated for each parameter pair. The independence was assessed by calculating the R-square value of the linear fit for each sample group. A low R-square indicates a poor fit and thereby a higher degree of independence. The pairwise scatter plots are shown in Fig. 6A–F. It can be seen from the analysis that Z - K and K - %R are the most independent pair of parameters, with the maximum R-square value of only 0.09 for the AN group in the K - %R plot. The k - %R pair was noted to be the least independent pair of parameters with R-square values of 0.48, 0.63, and 0.008 for the AN, FA, and CA samples. The lower degree of independence for the k - %R pair follows from the fact that both parameters are extracted from different aspects of the mechanical characterization of the samples. All the plots also show a clear separation between the AN and CA groups, with the FA group clustering between the two in a partially overlapped manner. A colormap of the R-square values for each of the three groups is shown in Fig. 6G–I. The colormaps indicate that the parameters are least independent for the FA samples, suggesting difficulty differentiating it from AN and CA, as was also evident from the experimental data. The Fisher’s combined probability test with all the four parameters in a multi-modal manner was able to differentiate between the three groups with the highest statistical significance. The test differentiated AN from FA, AN from CA, and CA from FA with *p* = 1.68e-9, *p* = 5.76e-13, and *p* = 4.5e-3 respectively. The*p* values for the tests with the other combinations of the parameters, including those for the single parameter Student t-tests for the three group comparisons, are summarized in Table 2. Since only Z was able to differentiate FA from CA and K, k, and %R was able to distinguish between AN and FA and AN and CA, combining Z with one of K, k, or %R was able to differentiate between all the three groups, albeit with lower statistical power. Additionally, though K or k alone was not able to distinguish CA from FA, testing with the combination of these two parameters was able to achieve the differentiation of CA from FA with a low degree of statistical significance. The data in the table, in summary, shows that since the parameters are fairly independent and add information to the differentiation, the p values progressively become lower as more parameters among the four are used as a basis for differentiation between the sample groups.

**Table 2:** Analysis of combination of modalities

**Modality**	**COMPARISON GROUP**		
	**AN vs. FA**	**AN vs. CA**	**FA vs. CA**
*Z*	1.65e-1 (ns)	2.6e-3 (**)	2.02e-2 (*)
*K*	6.4e-5 (***)	7.64e-8 (***)	1.15e-1 (ns)
*k*	1.6e-4 (***)	1.4e-4 (***)	8.1e-2 (ns)
*%R*	2.3e-4 (***)	2.2e-3 (**)	7.9e-2 (ns)
*Z and K*	1.32e-4 (***)	4.63e-9 (***)	1.64e-2 (*)
*Z and k*	3.05e-4 (***)	5.76e-6 (***)	1.21e-2 (*)
*Z and %R*	4.24e-4 (***)	7.5e-5 (***)	1.2e-2 (*)
*K and k*	2e-7 (***)	2.8e-10 (***)	5.3e-2 (ns)
*K and %R*	2.8e-7 (***)	4e-9 (***)	5.18e-2 (ns)
*k and %R*	6.7e-7 (***)	5e-6 (***)	3.9e-2 (*)
*Z, K, and k*	3.8e-7 (***)	1.44e-11 (***)	8.7e-3 (**)
*Z, K, and %R*	5.3e-7 (***)	1.9e-10 (***)	8.6e-3 (**)
*K, k, and %R*	9e-10 (***)	1.23e-11 (***)	2.52e-2 (*)
*Z, k, and %R*	1.2e-6 (***)	1.9e-7 (***)	6.5e-3 (**)
*Z, K, k, and %R*	1.68e-9 (***)	5.76e-13 (***)	4.5e-3 (**)

*0.01 < *p* < 0.05

**0.001 < *p* < 0.01

***p < 0.001

### Evaluation of classification accuracy using a gaussian process classifier

**Fig. 7:**
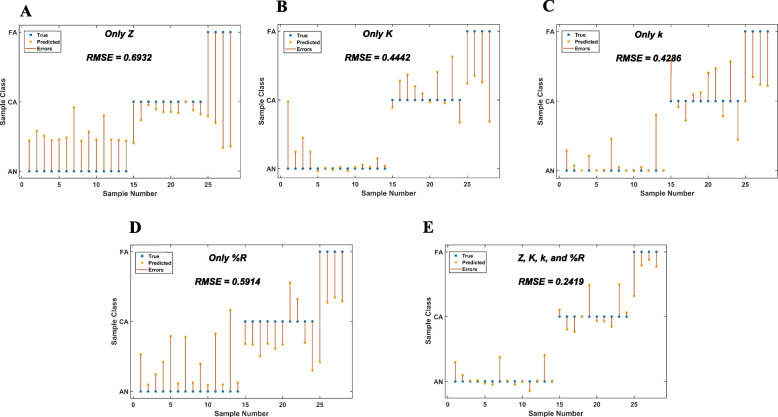
Evaluation of prediction errors with the gaussian process classifier using (**A**) only Z, (**B**) only K, (**C**) only k, (**D**) only percentage relaxation (%R), and (**E**) all four parameters for training the classifier

While Fisher’s combined *p*-value analysis only gives insights into the potential of the combination of parameters to better differentiate between the sample groups, it does not however perform any classification or assess its accuracy. For this, Gaussian process classifiers with different covariance kernels, namely, rational quadratic, squared exponential, exponential, and matern 5/2 were evaluated with the four biophysical parameters (Z, K, k, and %R) as input features to classify the samples into the three sample groups (AN, FA, and CA). The classification was performed using the leave-one-out cross-validation (LOOC) technique. The matern 5/2 kernel was overall found to be the best performing in terms of the RMSE. Figure 7 summarizes the key results from the Gaussian process classification using the matern 5/2 covariance kernel. The four parameters (Z, K, k, and %R) standalone and in various combinations were used as input features for the classification. Figure 7A–D shows the classification response plots along with the RMSE when each of the parameters Z, K, k, and %R alone was used as the metric for classification. Using Z as the feature gives the poorest RMSE of 0.6932. Using only K or k gives a better RMSE of 0.4442 and 0.4286, respectively, as compared to Z (0.6932) and %R (0.5914). However, the FA group has been significantly misclassified or poorly classified in all four cases, contributing significantly to the higher RMSE. The lowest RMSE of 0.2419 is obtained when all the four parameters are used as input features for the classification task (Fig. 7E). Using all the four parameters was also able to bring down the error in the classification of the FA group. Table S2 summarizes the RMSE values obtained for all the combinations of the parameters for the four different covariance kernels used for the classification. These results further corroborate the highly significant *p*-values obtained using the Fisher’s combined *p*-value tests with all the parameters.

## Discussion

The experimental setup demonstrated in this study with the system of biochips enables a comprehensive biophysical characterization of the breast tissue samples. The multiple changes that occur during tumourigenesis and tumour progression affect the tissue’s bulk electrical, thermal, and mechanical properties, be it benign or malignant, as is revealed through the experimental data presented. Histologically, the fibroadenoma is characterized by cell proliferation in both the stromal and epithelial components (biplastic) of lobules, with the glands and ducts growing over them to form the tumour mass. It is also observed to be hypo-vascular [20]. The malignant breast tumours (in this case, invasive ductal carcinoma), on the contrary, are a site of a more intense cellular proliferation, disorder, and leaky vasculature owing to a hypervascular stroma [21–23]. The extracellular matrix (ECM) and its transformations also play a key role in tumour progression [16, 24]. The two key components of the ECM, namely, the interstitial matrix and the basement membrane, undergo different degrees of transformation in benign and malignant tumours. While the remodelling of the interstitial matrix is observed in both benign and malignant cases, the transformation of the basement membrane is a unique characteristic of malignancy [25]. This remodelling of the basement membrane enables the cancer cells to invade the stromal tissue making the tumour mass malignant [26]. The remodelling of the ECM involves the processes of ECM deposition, chemical modifications, degradation by proteolysis, and force mediated transformations of the fibres which opens up passages for cells to invade and migrate. The ECM deposition process alters the abundance and composition of the components of the normal ECM. While malignancy alters all the four processes, benign tumours usually undergo only the ECM deposition with a few chemical modifications [27]. These transformations at the cellular and microenvironment levels are likely to be the key drivers of the differences in the biophysical properties between normal, benign, and malignant breast tissues.

The significantly higher mean impedance observed for the CA group compared to FA and AN is thus likely due to the modified and disrupted ECM and high cellular proliferation. The hyper-vascular stroma, which creates leaky vasculatures, makes it comparatively rich in water content. While the increased water content aids the electrical conduction in the in vivo and freshly excised ex vivo conditions through ionic transport, upon formalin fixation, this water content becomes electrically and thermally immobile. This, combined with the disrupted and modified ECM, makes the CA samples electrically less conductive than FA and AN. The modelled circuit parameters also reflect these changes. While the higher value of R_e_ obtained for CA reflects the changes in the disrupted ECM, the higher R_i_ captures the increased nuclear-cytoplasm ratio, higher genetic materials, and multinucleation observed in cancer cells. Though it is reported that cancer cells have a lower membrane capacitance than benign and normal, the higher C_m_ obtained for the CA group could be the cumulative sum of the membrane capacitances of the higher number of proliferated cells per unit volume of the tissue [28, 29].

With regard to the mechanical characterization, the higher stiffness in CA and FA samples can be attributed to the basal level ECM remodelling that occurs commonly in both groups through ECM deposition and alteration in the composition and abundance of the matrix fibre proteins, as discussed earlier. While the cells become softer, the matrix becomes stiffer owing to the matrix remodeling, leading to an overall stiffer tissue in the case of tumours (both benign and malignant) [30–32]. It has been shown through indentation and imaging studies that while elasticity and stiffness could be used to detect tumours, they most often lack the sensitivity to differentiate between benign and malignant cases due to an overlap seen in the measured values in the two groups [33, 34]. Additionally, formalin fixation reduces the overall sensitivity owing to the physical changes that accompany the fixation process [35]. This explains the overlap seen in the indentation curves for the CA and FA groups. On the contrary, viscoelastic relaxation has been reported to be a good indicator in differentiating between benign and malignant tumours [36–38]. The proteolytic degradation of the matrix seen in malignant tumours increases their degree of anisotropy compared to benign tumours [39]. Additionally, the increased ECM deposition with reduced degradation makes the benign tumours more isotropic than malignant and normal tissue. As a result, the benign tumours (FA) have a greater percentage of relaxation in load than CA and AN, as observed in this study.

The results obtained for the thermal conductivity of the samples follow the same biophysical and biochemical changes. The degraded and disrupted ECM and the poor thermal conductivity of the ECM fibres make the CA samples the least thermally conductivity, followed by FA and AN. The formalin fixation also impedes the thermal conductivity of the water content in the tissues making the overall thermal conductivity of all the sample groups lower than those reported in vivo and from freshly excised samples [40, 41]. Thus, the electrical, thermal, and mechanical characterization of the tissues help to capture the transformations that happen in the tissue microenvironment to serve as a basis to delineate between normal, benign, and malignant cases.

The Fisher’s combined probability test, which was used to assess the utility of using a combination of parameters, has traditionally been used in meta-analysis to combine data from independent studies to evaluate the statistical power of a hypothesis [42]. The same methodology has been adopted to combine the data from the different modalities in our experiment to assess the statistical power of a combined analysis. The classification using the GP method further corroborates the results obtained from the combined statistical analysis. The uncertainty estimates graphically depicted in the GP classification results help one to make an informed choice, especially for a clinical use case. Even though the FA group has a relatively smaller sample size (*N* = 4), the reduced RMSE obtained with all the four biophysical parameters as input features demonstrate the utility of the methodology to differentiate between the three sample groups. The higher RMSE obtained for the classification with the individual parameters, especially for the FA group, also shows that the classification is not an overestimation. The use of additional parameters to classify indeed helps pool the unique physiologically relevant information captured by those parameters.

The engineering technology, experimental setup, and results presented in this work propose a methodology using micro-engineered biochips and electronic system engineering to capture the biophysical phenotype of the specimen tissues. The use of microfabrication technology enables the measurement from smaller-sized samples (< 5 mm) that are often extracted during biopsies and surgical procedures. The technique is label-free and involves minimal sample preparation other than the ones done for routine diagnosis. The measurement of each sample takes 18 ± 2 min. Routine histopathology, though highly sensitive, takes 48 – 72 h to give the results. Frozen section examination, used during intra-operative margin assessment, takes about 20 min per sample but is skill-intensive and subjective, requiring a high level of expertise from the pathologist, who also uses a high suspicion index to arrive at the conclusions [43]. While several technologies have been developed for non-invasive diagnostic breast imaging for identifying cancerous lesions and for intra-operative margin identification from large-sized excised ex vivo samples during surgical resection, advancements toward improving the analysis and diagnosis of ex vivo tissues extracted during biopsy (such as samples from core needle (CNB) and incisional biopsy) have been limited [44, 45]. Most of the technologies available for diagnosing breast cancer from ex vivo tissues are compatible with analyzing only the larger-sized samples greater than 1 cm owing to the size of the equipment, the form factor of the probe, spatial resolution limitation, and power constraints [46–49]. Though there have been significant advancements in MEMS technology in developing intricate and innovative sensor designs, the system-level integration of these devices into product-looking prototypes useful for clinical testing has been limited. The MEMS devices that have seen success in human sample testing and clinical trials have primarily focused on the mechanical characterization of the tissues for applications such as in vivo assessment or guided tissue targeting [50]. Devices designed for electrical and thermal characterization have been primarily used for monitoring cell cultures or assessing differences between cancerous and normal cells in culture plates [51–55]. MEMS devices integrated on portable systems, leveraging their advantage of miniaturization, to comprehensively assess the multimodal biophysical phenotype of small-sized biopsy tissue samples have not been reported. The system reported in this work has been specifically designed to supplement this technology gap by providing a solution for comprehensive biophysical analysis of the tissue samples in the form factor of a portable table-top semi-automated setup integrated with biochips in the pathology laboratories to aid the diagnostic workflow. The system can be used not only for analyzing breast cancer biopsy tissues but also for other cancers that form solid tumours, such as oral, cervical, pancreatic cancers, etc., after sufficient experiments to understand the baseline values of the normal tissues in which such cancers originate.

From a technology perspective, the system and methodology provide only bulk information on the electrical, thermal, and mechanical characteristics of the sample. It does not perform imaging nor provide micro-structural information about the tissue architecture. The current form factor of the system limits its direct adoption for an in vivo application, such as for intra-operative margin assessment and surgical guidance. The biochip, indenters, and tissue holder have been designed to probe an ex vivo sample and measure its bulk properties. Additionally, owing to the finite dimensions of the edges of the indenter and the sensor-indenter interface module, samples less than 1.5 mm in thickness cause the two arms of the system to touch each other, leading to unreliable contact with the sample. For the sample dimensions greater than 7 mm in thickness, the thermal testing results in considerable time to reach equilibrium. Additionally, the electrical charge penetration gets affected at higher thicknesses. Owing to the above practical and theoretical limitations, the sample dimensions were kept above 1.5 mm and below 7 mm during the processing. The dimensions of the system (205 mm x 310 mm x 165 mm (L X B X H)) also restrict its use in an in vivo application. However, the modalities (ETM) discussed and statistical analysis methods could be adopted when designing a system for in vivo studies in the future.

## Conclusions

The study presents a unique methodology using micro-engineered biochips and electronic system engineering to capture the biophysical phenotype of tissues. The electrical, thermal, and mechanical properties of the tissue characterized through the measurement of the electrical impedance, thermal conductivity, mechanical stiffness, and viscoelastic relaxation help to comprehensively characterize the different constituent elements of the tissue to serve as a basis for delineation between normal, benign, and malignant breast biopsy tissues. Measurements from *N *= 14 formalin-fixed paired tumour and adjacent normal breast biopsy tissues show that a combination of all four parameters can differentiate and classify all the three sample groups with good statistical significance (*p* < 4.5e-3) and least root mean square error (0.2419), respectively. While formalin-fixation is a routine procedure used in pathology laboratories for tissue preservation and analysis, the same methodology can be applied to freshly excised tissues stored in saline solution. While the current methodology could be readily adapted for use in a pathology laboratory, studies with freshly excised tissues could pave the way for its potential application in operating rooms during surgery. We envisage integrating this methodology on a hand-held probe for its application in in vivo tumour delineation during surgical procedures and for other applications which require soft tissue analysis.

## Methods

### Fabrication of the SoB

S1, the biochip for force sensing, is fabricated using a five-mask process on a 3-inch oxidized silicon wafer. The oxide layer is patterned using photolithography followed by boron diffusion to create the four piezoresistive elements around the chip. Subsequent window opening and diffusion steps create the p + contacts. Titanium/Gold (15 nm/140 nm) is then deposited and patterned to create the contacts for the piezoresistive bridges. These bridges are positioned at the edges of a diaphragm of thickness 30 μm, created using bulk micromachining of the silicon substrate using deep reactive ion etching, which constitutes the last mask in the process. Each chip is then diced from the wafer using automatic dicer to realize the device. This microforce sensor is a design variant of a previously reported sensor from our group with changes in the bridge structure of the piezoresistive elements [56]. A two-mask process is used to fabricate S2 and S3, the biochips for electro-thermal sensing. The functional pattern is created as an array of biochips on a 3-inch oxidized silicon wafer by depositing and patterning Titanium/Platinum (30/150 nm) using a lift-off process. Photolithography with the second mask followed by deep reactive ion etching for bulk micromachining creates a thermal isolation trench around the microheater. The biochips are then diced from the silicon wafer using the automatic dicer to realize the devices.

### Details of samples used for the study

The ETM characterization was performed on formalin-fixed excisional breast biopsy samples from *N* = 14 subjects ((ethics approval from Assam Medical College (Ref. No: AMC/EC/1334) and biosafety (Ref. No: IBSC/IISc/HP/01/2019) and human ethics (Ref. No: 05/582,021) committee approval from the Indian Institute of Science). From the samples excised for a routine examination, the pathologist extracted a part of the tumour and adjacent normal tissue for the study. Three types of breast tissue: fibroadenoma (benign), invasive carcinoma (malignant), and adjacent normal, were used for the study. The pathologist classified the samples into each group by microscopic examination of the histopathology as part of the routine hematoxylin and eosin staining for each tumour. Tumour samples from *N* = 10 subjects diagnosed with invasive ductal carcinoma (CA) and *N* = 4 subjects of type fibroadenoma (FA) were used for the study, with the corresponding adjacent normal tissue. Uniform cuboidal blocks of tumour and adjacent normal tissues with dimensions 4 ± 0.12 mm x 4 ± 0.18 mm x 4 ± 0.15 mm were prepared using a surgical knife from the extracted samples and stored in separate 1.5 mL tubes filled with 10% buffered formalin. At the time of measurement, the samples were removed from the formalin tubes and loaded into the system for the ETM characterization.

### Data analysis and statistical testing

The electrical impedance ($Z$) from 10 Hz to 3 MHz at a tissue temperature of 37 ℃, the tissue temperature at the sensing (to assess the thermal energy transmitted through the sample) and source (microheater) side as a function of microheater temperature, and the mechanical loading and stress-relaxation data were captured from the *N* = 14 paired adjacent normal and tumour (FA and CA) samples. Four key biophysical parameters, namely, the impedance value, $Z$ at 15 kHz, the thermal conductivity, $K$ at 37 ℃, the mechanical stiffness, $k$ at 20% strain, and the percentage relaxation in normalized load, $\%R$ after 150 s of stress-relaxation were then extracted from the raw experimental data. These biophysical parameters formed the basis for the subsequent statistical tests to assess the differences between the sample groups (AN, FA, and CA). The Student’s t-test assuming unequal variance (Welch’s correction) was used to assess the statistical differences between the three sample groups for each of the four biophysical parameters. Before applying the Student’s t-test, the data from each group were confirmed to follow the normal distribution using the Shapiro-Wilk test.

While the Student’s t-test is useful in understanding how each of the four parameters is different across AN, FA, and CA, we also wanted to see whether a combination of these parameters can be used to differentiate between the sample groups with a higher statistical significance. The Fisher’s combined probability test was used to evaluate the significance level of the differentiation between the sample groups when a combination of the biophysical parameters was used as a metric for delineation. The Fisher’s test combines *p*-values from independent tests bearing the same overall null hypothesis, and the combined statistic is known to follow the chi-squared distribution [57, 58]. To ensure that combined *p*-values are not over-estimated, the independence of the parameters with respect to each other was evaluated by calculating the R square value of pairwise scatter plots of the parameter values for the three groups. A low R-square value indicates poor goodness of fit and correlation and thus a higher level of independence between the parameters.

As a final step in the statistical analysis pipeline, the gaussian process (GP) method was used to classify the samples as AN, FA, or CA using each of the four parameters and combining them as input features. The accuracy of the classification was evaluated using the root mean square error (RMSE) as a metric. GP is a non-parametric supervised learning method that models a Gaussian distribution over unknown functions and has been used for regression and classification tasks [59]. GPs thus extent multi-variate Gaussian distributions to random functions with probability distributions that follow the Gaussian curve. The key components of this distribution of unknown functions are the mean and covariance kernel functions, which can fully describe the GP. Such a non-parametric probabilistic classification method also considers the uncertainty of the predictions, which becomes useful in clinical settings by providing an option of rejecting uncertain cases and a potential application of decision theories to optimize the classification in the future [60]. GPs have been used for classification in several biomedical applications, such as estimation of Parkinson’s disease based on speech, classification of Alzheimer’s disease patients, classification and grading of histological images from prostate and breast cancer patients, to name a few [61–65]. Though widely applied for regression tasks, the less-explored aspect of using the GPs for classification with uncertainty estimates has been utilized in our study.

### Supplementary Information

**Additional file 1: Fig. S1.** Plots of the magnitude of impedance (A) – (N) for 14 sample pairs. **Fig. S2.** Plot of the mean (A) phase response and (B) real part of impedance vs. imaginary part for the AN, FA, and CA samples. **Fig. S3.** Modified Cole-Cole model of the tissue used for fitting the experimental impedance data to obtain the circuit parameters. **Table S1.** Values of the fitted circuit parameters for the three sample groups (AN, FA, and CA) for the modified Cole-Cole model. **Fig. S4.** Experimental and fitted plots for extracting circuit parameters from the mean impedance magnitude curves of (A) AN, (B) FA, and (C) CA samples. **Fig. S5.** Experimental and fitted plots from the mean phase data curves of (A) AN, (B) FA, and (C) CA samples. **Fig. S6.** (A) – (N) Plots of the mechanical loading characteristics for 14 sample pairs. **Table S2.** RMSE values obtained for the different combinations of input features from Z, K, k, and %R with the different gaussian process covariance kernels. 

** Additional file 2.**

## Data Availability

The data that supports the findings of the study are available from the corresponding author upon reasonable request.

## References

[1] Sung H, Ferlay J, Siegel RL, Laversanne M, Soerjomataram I, Jemal A (2021). Global Cancer Statistics 2020: GLOBOCAN Estimates of Incidence and Mortality Worldwide for 36 Cancers in 185 Countries. CA Cancer J Clin.

[2] Saadatmand S, Bretveld R, Siesling S, Tilanus-Linthorst MMA (2015). Influence of tumour stage at Breast cancer detection on survival in modern times: population based study in 173 797 patients. BMJ.

[3] Pilewskie M, Morrow M (2018). Margins in Breast Cancer: how much is Enough?. Cancer.

[4] Nowikiewicz T, Śrutek E, Głowacka-Mrotek I, Tarkowska M, Żyromska A, Zegarski W (2019). Clinical outcomes of an intraoperative surgical margin assessment using the fresh frozen section method in patients with invasive Breast cancer undergoing breast-conserving Surgery – a single center analysis. Sci Rep.

[5] de CHOLNOKY T. BENIGN TUMORS OF THE BREAST (1939). Arch Surg.

[6] Guray M, Sahin AA (2006). Benign breast Diseases: classification, diagnosis, and management. Oncologist.

[7] Bedrosian I, Mick R, Orel SG, Schnall M, Reynolds C, Spitz FR (2003). Changes in the surgical management of patients with breast carcinoma based on preoperative magnetic resonance imaging. Cancer.

[8] Shipp DW, Rakha EA, Koloydenko AA, Macmillan RD, Ellis IO, Notingher I (2018). Intra-operative spectroscopic assessment of surgical margins during breast conserving Surgery. Breast Cancer Res.

[9] Fletcher SW (2011). Breast cancer screening: a 35-year perspective. Epidemiol Rev.

[10] Pal UM, Saxena M, Anil Vishnu GK, Parsana D, Sarvani BS, Varma M, Jayachandra M, Kurpad V, Baruah D, Gogoi G, Vaidya JS. Optical spectroscopy-based imaging techniques for the diagnosis of breast cancer: A novel approach. Appl Spectrosc Rev. 2020;55(8):778–804.

[11] Damez J-L, Clerjon S (2008). Meat quality assessment using biophysical methods related to meat structure. Meat Sci.

[12] Dutta D, Palmer X-L, Ortega-Rodas J, Balraj V, Dastider IG, Chandra S (2020). Biomechanical and Biophysical Properties of Breast Cancer cells under varying glycemic regimens. Breast Cancerï¿½(Auckl).

[13] Tracqui P (2009). Biophysical models of tumour growth. Rep Prog Phys.

[14] Małecka-Massalska T, Chara K, Gołębiowski P, Władysiuk M, Smoleń A, Kurylcio A (2013). Altered tissue electrical properties in women with Breast cancer–preliminary observations. Ann Agric Environ Med.

[15] Gautherie M (1980). Thermopathology of Breast cancer: measurement and analysis of in vivo temperature and blood flow. Ann N Y Acad Sci.

[16] Lu P, Weaver VM, Werb Z (2012). The extracellular matrix: a dynamic niche in cancer progression. J Cell Biol.

[17] Vishnu GKA, Sakorikar T, Baby A, Singh C, Rangarajan A, Pandya HJ (2021). Bimodal characterization of breast biopsy tissues using MEMS-Based biochips: toward Improved Tumor Delineation. IEEE Sens J.

[18] Av GK, Gogoi G, Behera B, Rila S, Rangarajan A, Pandya HJ (2022). RapidET: a MEMS-based platform for label-free and rapid demarcation of tumors from normal breast biopsy tissues. Microsyst Nanoeng.

[19] Leikina E, Mertts MV, Kuznetsova N, Leikin S (2002). Type I collagen is thermally unstable at body temperature. Proc Nat Acad Sci.

[20] Ajmal M, Khan M, Van Fossen K. Breast Fibroadenoma. StatPearls [Internet]. Treasure Island (FL): StatPearls Publishing; 2022. Available from: http://www.ncbi.nlm.nih.gov/books/NBK535345/. [cited 2022 Apr 22]. 30570966

[21] Hanahan D (2022). Hallmarks of Cancer: New dimensions. Cancer Discov.

[22] Hanahan D, Weinberg RA (2011). Hallmarks of Cancer: the Next Generation. Cell.

[23] Mankoff DA, Dunnwald LK, Gralow JR, Ellis GK, Charlop A, Lawton TJ, Livingston RB. Blood flow and metabolism in locally advanced breast cancer: relationship to response to therapy. J Nucl Med. 2002:43(4):500–9. 11937594

[24] Winkler J, Abisoye-Ogunniyan A, Metcalf KJ, Werb Z (2020). Concepts of extracellular matrix remodelling in tumour progression and Metastasis. Nat Commun.

[25] Egeblad M, Rasch MG, Weaver VM (2010). Dynamic interplay between the collagen scaffold and Tumor evolution. Curr Opin Cell Biol.

[26] Kessenbrock K, Plaks V, Werb Z (2010). Matrix metalloproteinases: regulators of the Tumor Microenvironment. Cell.

[27] Kai F, Drain AP, Weaver VM (2019). The Extracellular Matrix modulates the metastatic journey. Dev Cell.

[28] Morimoto T, Kimura S, Konishi Y, Komaki K, Uyama T, Monden Y (1993). A study of the electrical bio-impedance of tumors. J Invest Surg.

[29] Pandya HJ, Kim HT, Roy R, Chen W, Cong L, Zhong H (2014). Towards an automated MEMS-based characterization of benign and cancerous breast tissue using bioimpedance measurements. Sens Actuators B.

[30] Emon B, Bauer J, Jain Y, Jung B, Saif T (2018). Biophysics of Tumor Microenvironment and Cancer Metastasis - A Mini Review. Comput Struct Biotechnol J.

[31] Leight JL, Drain AP, Weaver VM (2017). Extracellular matrix remodeling and stiffening modulate Tumor phenotype and treatment response. Annual Rev Cancer Biology.

[32] Walker C, Mojares E, del Río Hernández A. Role of Extracellular Matrix in Development and Cancer Progression. Int J Mol Sci. 2018 ;19. Available from: https://www.ncbi.nlm.nih.gov/pmc/articles/PMC6213383/.[cited 2020 Nov 19]. 10.3390/ijms19103028 PMC6213383 30287763

[33] Goenezen S, Dord J-F, Sink Z, Barbone PE, Jiang J, Hall TJ (2012). Linear and nonlinear elastic modulus imaging: an application to Breast cancer diagnosis. IEEE Trans Med Imaging.

[34] Sigrist RMS, Liau J, Kaffas AE, Chammas MC, Willmann JK (2017). Ultrasound Elastography: review of techniques and clinical applications. Theranostics.

[35] Werner M, Chott A, Fabiano A, Battifora H (2000). Effect of formalin tissue fixation and processing on immunohistochemistry. Am J Surg Pathol.

[36] Bayat M, Nabavizadeh A, Kumar V, Gregory A, Insana M, Alizad A (2018). Automated in vivo Sub-hertz Analysis of Viscoelasticity (SAVE) for evaluation of breast lesions. IEEE Trans Biomed Eng.

[37] Madani N, Mojra A (2017). Quantitative diagnosis of breast tumors by characterization of viscoelastic behavior of healthy breast tissue. J Mech Behav Biomed Mater.

[38] Zhang H, Wang Y, Insana MF (2016). Ramp-hold relaxation solutions for the KVFD model applied to soft viscoelastic media. Meas Sci Technol.

[39] Sinkus R, Tanter M, Catheline S, Lorenzen J, Kuhl C, Sondermann E (2005). Imaging anisotropic and viscous properties of breast tissue by magnetic resonance-elastography. Magn Reson Med.

[40] Lozano A, Hayes JC, Compton LM, Azarnoosh J, Hassanipour F (2020). Determining the thermal characteristics of Breast cancer based on high-resolution infrared imaging, 3D breast scans, and magnetic resonance imaging. Sci Rep.

[41] Valvano JW. Tissue thermal properties and perfusion. Optical-thermal response of laser-irradiated tissue. Springer; 1995. pp. 445–88.

[42] Yoon S, Baik B, Park T, Nam D (2021). Powerful p-value combination methods to detect incomplete association. Sci Rep.

[43] Jaafar H (2006). Intra-operative Frozen Section Consultation: concepts, Applications and limitations. Malays J Med Sci.

[44] Kamal AMohd, Sakorikar T, Pal UM, Pandya HJ. Engineering approaches for Breast Cancer diagnosis: a review. IEEE Rev Biomed Eng. 2022;1–21. 10.1109/RBME.2022.3181700 35687618

[45] Pradipta AR, Tanei T, Morimoto K, Shimazu K, Noguchi S, Tanaka K (2020). Emerging technologies for Real-Time intraoperative Margin Assessment in Future breast-conserving Surgery. Adv Sci.

[46] Zhang Z, Pei J, Wang D, Gan Q, Ye J, Yue J (2016). A wearable Goggle navigation system for dual-mode optical and ultrasound localization of suspicious lesions: validation studies using tissue-simulating phantoms and an ex vivo human breast tissue model. PLoS ONE.

[47] Moschetta M, Telegrafo M, Introna T, Coi L, Rella L, Ranieri V (2015). Role of specimen US for predicting resection margin status in breast conserving therapy. G Chir.

[48] Kaufman CS, Jacobson L, Bachman BA, Kaufman LB, Mahon C, Gambrell L-J (2007). Intraoperative digital specimen mammography: rapid, accurate results expedite Surgery. Ann Surg Oncol.

[49] Agresti R, Trecate G, Ferraris C, Valeri B, Maugeri I, Pellitteri C (2013). Ex vivo MRI evaluation of breast tumors: a novel tool for verifying resection of nonpalpable only MRI detected lesions. Breast J.

[50] Yu X, Wang H, Ning X, Sun R, Albadawi H, Salomao M (2018). Needle-shaped ultrathin piezoelectric microsystem for guided tissue targeting via mechanical sensing. Nat Biomedical Eng.

[51] Zhai J, Yi S, Jia Y, Mak P-I, Martins RP (2019). Cell-based drug screening on microfluidics. TRAC Trends Anal Chem.

[52] Tigli O, Bivona L, Berg P, Zaghloul ME (2010). Fabrication and characterization of a Surface-Acoustic-Wave Biosensor in CMOS Technology for Cancer Biomarker Detection. IEEE Trans Biomed Circuits Syst.

[53] Rajagopal MC, Valavala KV, Gelda D, Ma J, Sinha S (2018). Fabrication and characterization of thermocouple probe for use in intracellular thermometry. Sens Actuators A: Phys.

[54] Alexander F Jr, Price DT, Bhansali S. Optimization of interdigitated electrode (IDE) arrays for impedance based evaluation of Hs 578T cancer cells. Journal of Physics: Conference Series. IOP Publishing; 2010. p. 012134.

[55] Pradhan R, Mandal M, Mitra A, Das S (2014). Monitoring cellular activities of cancer cells using impedance sensing devices. Sens Actuators B.

[56] Alekya B, Sitaramgupta V, Vsn BSA, Pandya HJ (2022). Sensor for Meso-Scale tissue stiffness characterization. IEEE Sens J.

[57] Chen Z, Yang W, Liu Q, Yang JY, Li J, Yang MQ (2014). A new statistical approach to combining p-values using gamma distribution and its application to genome-wide association study. BMC Bioinformatics.

[58] Chen Z (2022). Optimal tests for combining p-Values. Appl Sci.

[59] Rasmussen CE. Gaussian Processes in Machine Learning. In: Bousquet O, von Luxburg U, Rätsch G, editors. Advanced Lectures on Machine Learning: ML Summer Schools 2003, Canberra, Australia, February 2–14, 2003, Tübingen, Germany, August 4–16, 2003, Revised Lectures [Internet]. Berlin, Heidelberg: Springer; 2004 [cited 2022 Apr 20]. p. 63–71. 10.1007/978-3-540-28650-9_4.

[60] Ashby D, Smith AF (2000). Evidence-based medicine as bayesian decision-making. Stat Med.

[61] Amaral T, McKenna SJ, Robertson K, Thompson A (2013). Classification and immunohistochemical scoring of breast tissue microarray spots. IEEE Trans Biomed Eng.

[62] Despotovic V, Skovranek T, Schommer C (2020). Speech Based Estimation of Parkinson’s Disease using gaussian processes and automatic relevance determination. Neurocomputing.

[63] Esteban ÁE, López-Pérez M, Colomer A, Sales MA, Molina R, Naranjo V (2019). A new optical density granulometry-based descriptor for the classification of prostate histological images using shallow and deep gaussian processes. Comput Methods Programs Biomed.

[64] Petinrin OO, Li X, Wong K-C (2022). Particle swarm optimized gaussian process classifier for treatment discontinuation prediction in Multicohort Metastatic Castration-resistant Prostate Cancer patients. IEEE J Biomedical Health Inf.

[65] Young J, Modat M, Cardoso MJ, Ashburner J, Ourselin S. Classification of Alzheimer’s disease patients and controls with Gaussian processes. 2012 9th IEEE International Symposium on Biomedical Imaging (ISBI). 2012. p. 1523–6.

