# Supplementary material for: Label-free multimodal electro-thermo-mechanical (ETM) phenotyping as a novel biomarker to differentiate between normal, benign, and cancerous breast biopsy tissues
Source: J Biol Eng. 2023 Nov 13;17:68. doi: 10.1186/s13036-023-00388-y (PMC10644568; doi:10.1186/s13036-023-00388-y)
Supplement: Supplementary file 1 — Additional file 1: Fig. S1. Plots of the magnitude of impedance (A) – (N) for 14 sample pairs. Fig. S2. Plot of the mean (A) phase response and (B) real part of impedance vs. imaginary part for the AN, FA, and CA samples. Fig. S3. Modified Cole-Cole model of the tissue used for fitting the experimental impedance data to obtain the circuit parameters. Table S1. Values of the fitted circuit parameters for the three sample groups (AN, FA, and CA) for the modified Cole-Cole model. Fig. S4. Experimental and fitted plots for extracting circuit parameters from the mean impedance magnitude curves of (A) AN, (B) FA, and (C) CA samples. Fig. S5. Experimental and fitted plots from the mean phase data curves of (A) AN, (B) FA, and (C) CA samples. Fig. S6. (A) – (N) Plots of the mechanical loading characteristics for 14 sample pairs. Table S2. RMSE values obtained for the different combinations of input features from Z, K, k, and %R with the different gaussian process covariance kernels. [file 13036_2023_388_MOESM1_ESM.pdf]

Supplementary Material

for

Label-free multimodal electro-thermo-mechanical (ETM) phenotyping as a novel biomarker to differentiate between normal, benign, and cancerous breast biopsy tissues

Anil Vishnu G K[1], Gayatri Gogoi[2], Midhun C. Kachappilly[3], Annapoorni Rangarajan[4] and Hardik J. Pandya[3,5,*]

AFFILIATIONS

[1]Center for BioSystems Science and Engineering, Indian Institute of Science, Bangalore, Karnataka 560012, India.

[2]Department of Pathology, Assam Medical College, Dibrugarh, Assam 786002, India.

[3]Department of Electronic Systems Engineering, Indian Institute of Science, Bangalore, Karnataka 560012, India.

[4]Department of Developmental Biology and Genetics, Indian Institute of Science, Bangalore, Karnataka 560012, India.

[5]Centre for Product Design and Manufacturing, Indian Institute of Science, Bangalore, Karnataka 560012, India.

[*]Author to whom correspondence should be addressed: Hardik J. Pandya (hjpandya@iisc.ac.in)

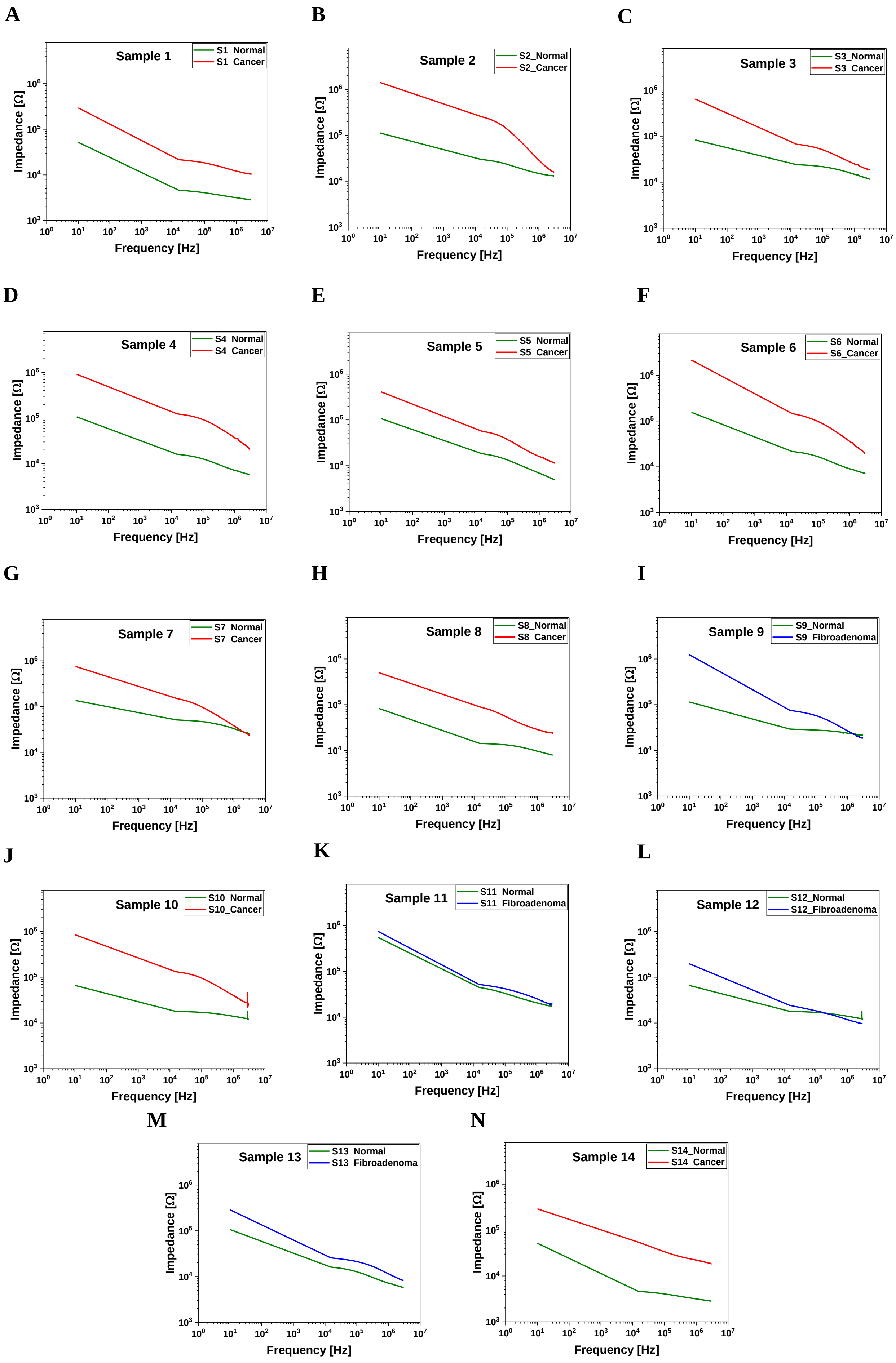

Fig. S1 Plots of the magnitude of impedance (**A**) – (**N**) for 14 sample pairs.

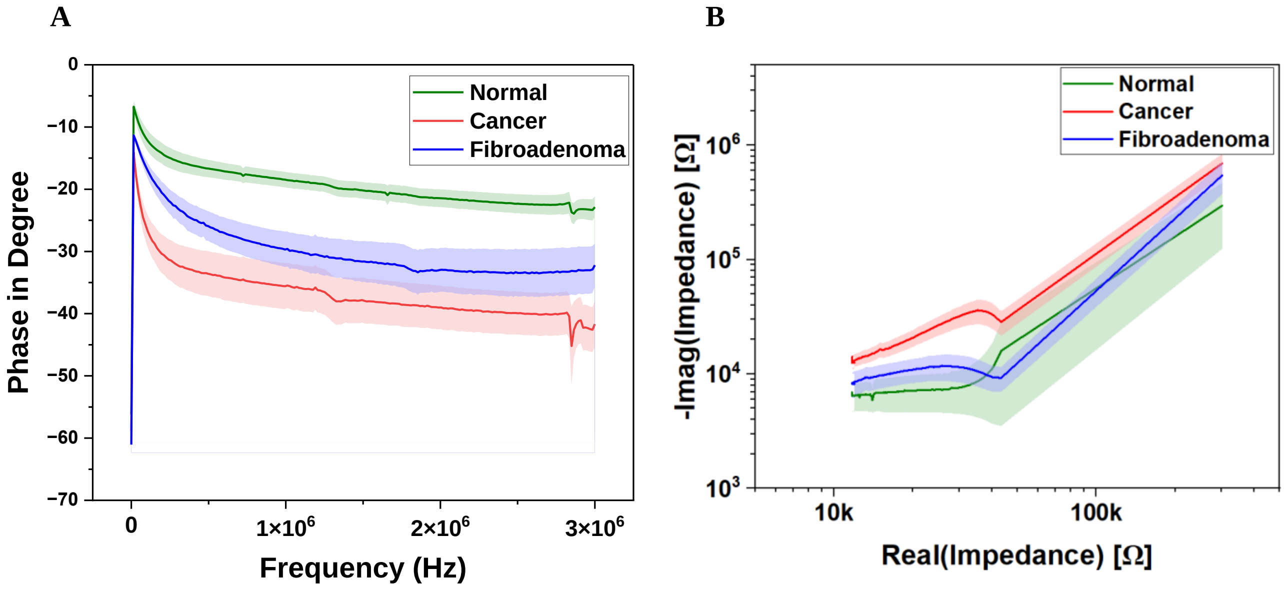

Fig. S2 Plot of the mean (**A**) phase response and (**B**) real part of impedance vs. imaginary part for the AN, FA, and CA samples.

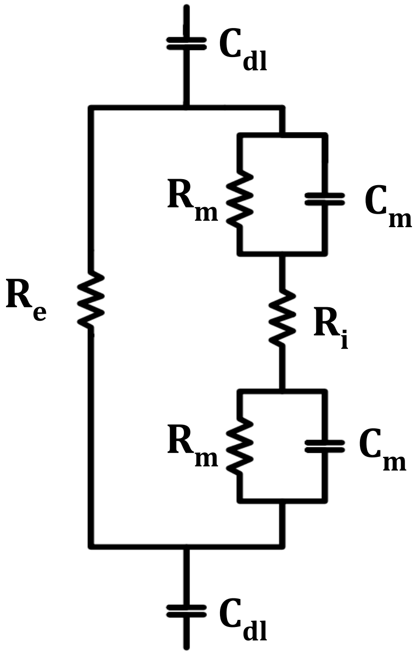

Fig. S3 Modified Cole-Cole model of the tissue used for fitting the experimental impedance data to obtain the circuit parameters.

Table S1 Values of the fitted circuit parameters for the three sample groups (AN, FA, and CA) for the modified Cole-Cole model.

Circuit Parameter	SAMPLE GROUP		
	**AN**	**FA**	**CA**
R_e (Ω)	2.27e4 ± 1.78e3	3.51e4 ± 4.34e3	1.75e5 ± 7.07e3
R_i (Ω)	1.23e4 ± 5.31e2	1.3e4 ± 2.33e2	3.29e4 ± 1.49e3
R_m (Ω)	9.27e4 ± 1.33e4	5.48e6 ± 1.14e5	6.43e4 ± 3.14e3
C_m (F)	1.1e-11 ± 1.63e-12	1.11e-11 ± 6.94e-13	1.02e-10 ± 1.77e-11
C_{dl} (F)	2.3e-7 ± 3.3e-8	5.28e-8 ± 7.8e-9	3.77e-8 ± 3.54e-9

A

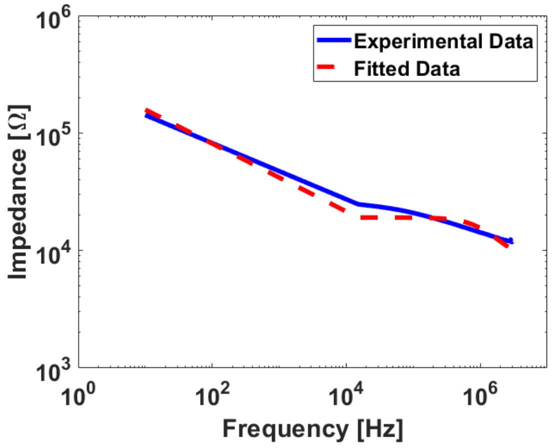

B

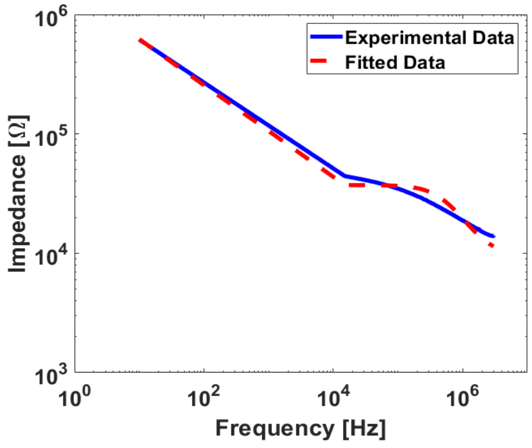

C

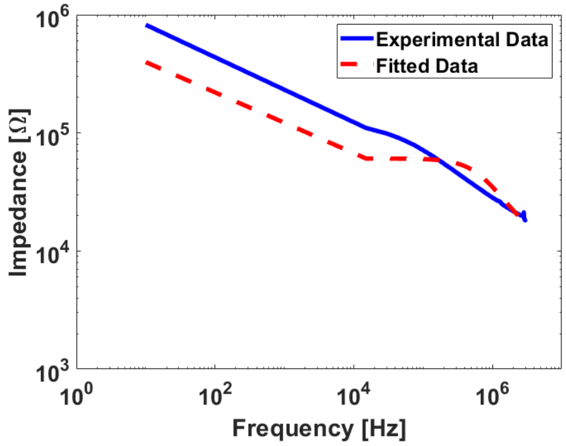

Fig. S4 Experimental and fitted plots for extracting circuit parameters from the mean impedance magnitude curves of (**A**) AN, (**B**) FA, and (**C**) CA samples.

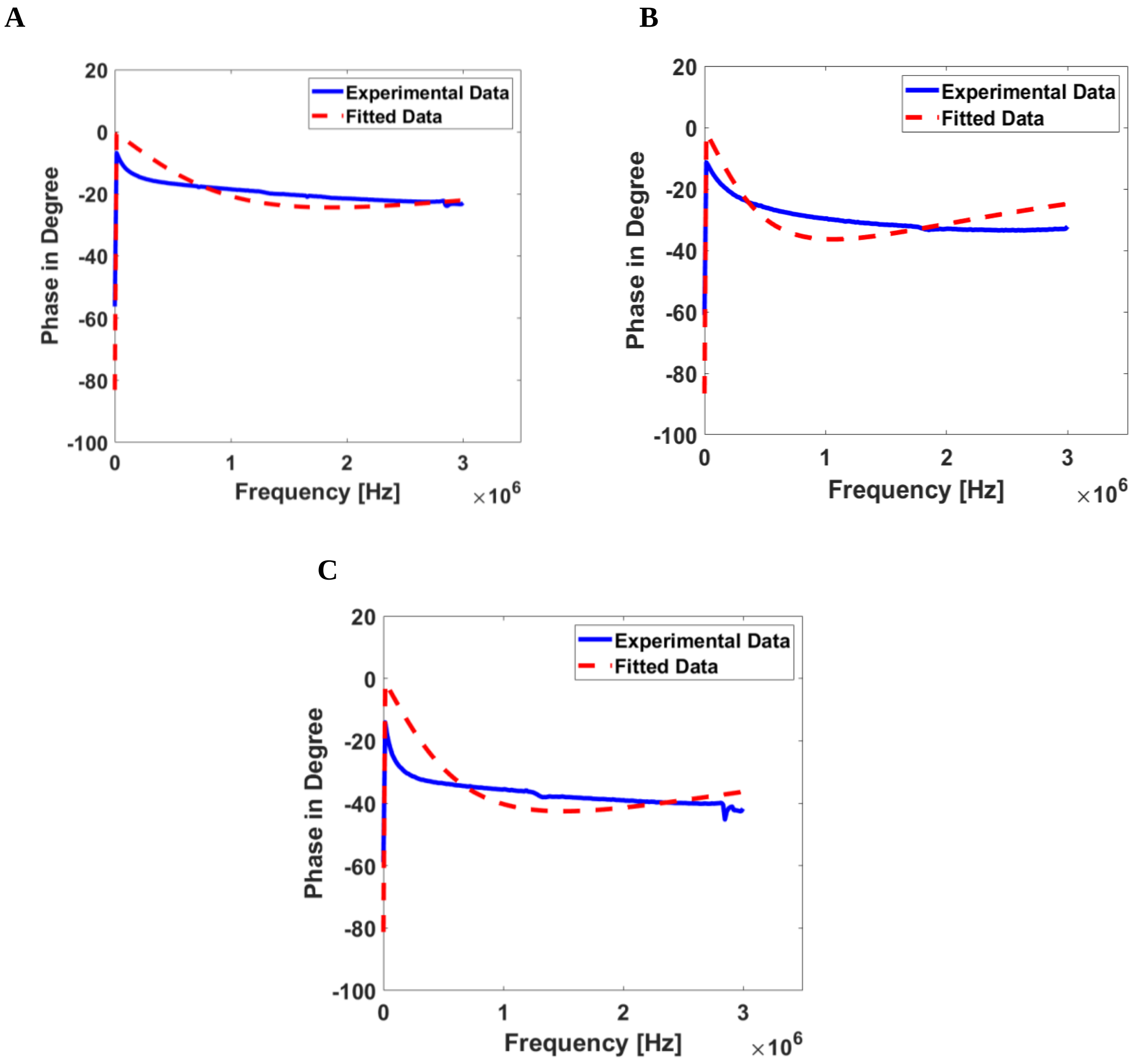

Fig. S5 Experimental and fitted plots from the mean phase data curves of (**A**) AN, (**B**) FA, and (**C**) CA samples.

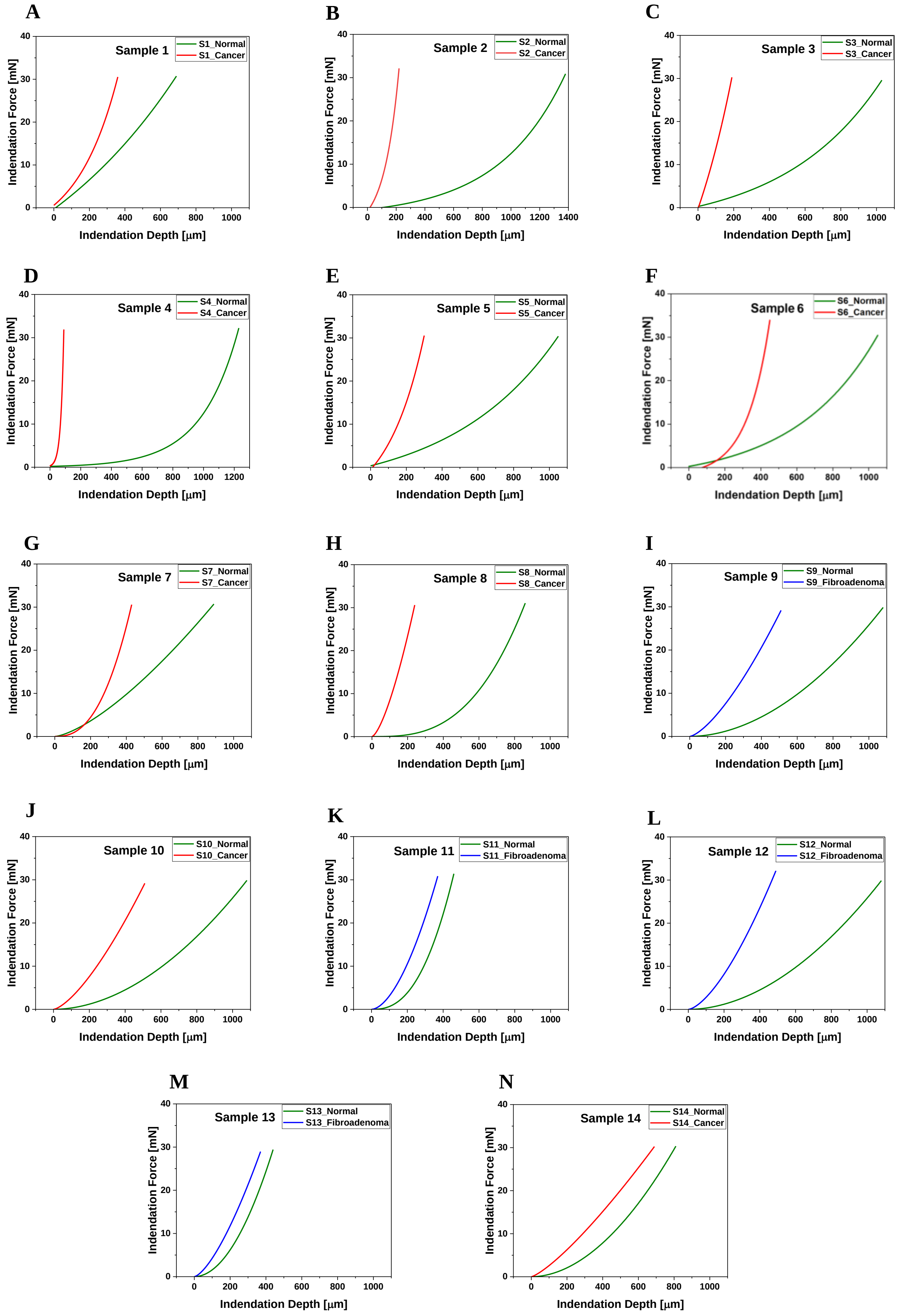

Fig. S6 (**A**) – (**N**) Plots of the mechanical loading characteristics for 14 sample pairs.

Table S2 RMSE values obtained for the different combinations of input features from Z, K, k, and %R with the different gaussian process covariance kernels.

INPUT FEATURE	RMSE OF CLASSIFICATION FOR DIFFERENT COVARIANCE KERNELS			
	**Rational Quadratic**	**Squared Exponential**	**Exponential**	**Matern 5/2**
$\boldsymbol{Z}$	0.6980	0.6980	0.7708	0.6932
$\boldsymbol{K}$	0.4391	0.4391	0.4747	0.4442
$\boldsymbol{k}$	0.4309	0.4308	0.4515	0.4286
$\%\boldsymbol{R}$	0.5917	0.5917	0.5927	0.5914
$\boldsymbol{Z\ and\ K}$	0.4834	0.4804	0.4732	0.4344
$\boldsymbol{Z\ and\ k}$	0.4089	0.4088	0.4355	0.4238
$\boldsymbol{Z\ and}\ \%\boldsymbol{R}$	0.4919	0.4894	0.4799	0.4661
$\boldsymbol{K\ and\ k}$	0.4112	0.4112	0.4340	0.4111
$\boldsymbol{K\ and}\ \%\boldsymbol{R}$	0.4890	0.4469	0.4787	0.4848
$\boldsymbol{k\ and}\ \%\boldsymbol{R}$	0.4768	0.4636	0.4717	0.4643
$\boldsymbol{Z, K, and\ k}$	0.3420	0.3608	0.3272	0.3088
$\boldsymbol{Z, K, and}\ \%\boldsymbol{R}$	0.3393	0.3309	0.3159	0.2936
$\boldsymbol{K, k, and}\ \%\boldsymbol{R}$	0.4205	0.4194	0.4266	0.4180
$\boldsymbol{Z, k, and}\ \%\boldsymbol{R}$	0.4392	0.4520	0.3883	0.4113
$\boldsymbol{Z, K, k, and}\ \%\boldsymbol{R}$	0.2480	0.2492	0.2935	0.2419
